# Occurrence and incidence rate of peripheral intravascular catheter-related phlebitis and complications in critically ill patients: a prospective cohort study (AMOR-VENUS study)

**DOI:** 10.1186/s40560-020-00518-4

**Published:** 2021-01-06

**Authors:** Hideto Yasuda, Ryohei Yamamoto, Yoshiro Hayashi, Yuki Kotani, Yuki Kishihara, Natsuki Kondo, Kosuke Sekine, Nobuaki Shime, Keita Morikane, Takayuki Abe, Toru Takebayashi, Mikihiro Maeda, Takuya Shiga, Taku Furukawa, Mototaka Inaba, Sachito Fukuda, Kiyoyasu Kurahashi, Sarah Murakami, Yusuke Yasumoto, Tetsuro Kamo, Masaaki Sakuraya, Rintaro Yano, Toru Hifumi, Masahito Horiguchi, Izumi Nakayama, Masaki Nakane, Kohei Ota, Tomoaki Yatabe, Masataka Yoshida, Maki Murata, Kenichiro Fujii, Junki Ishii, on behalf of the AMOR-VENUS study group

**Affiliations:** 1 Department of Emergency and Critical Care Medicine, Jichi Medical University Saimata Medical Center, 1-847, Amanuma-cho, Oomiya-ku, Saitama-shi, Saitama, 330-8503 Japan; 2 Department of Clinical Research Education and Training Unit, Keio University Hospital Clinical and Translational Research Center (CTR), Tokyo, Japan; 3 Department of Preventive Medicine and Public Health, Keio University School of Medicine, Tokyo, Japan; 4 Department of Intensive Care Medicine, Kameda Medical Center, Chiba, Japan; 5 Department of Critical Care Medicine, Japanese Red Cross Society Wakayama Medical Center, Wakayama, Japan; 6 Emergency and Critical Care Medicine, Japanese Red Cross Musashino Hospital, Tokyo, Japan; 7 Department of Medical Engineer, Kameda Medical Center, Chiba, Japan; 8 Department of Emergency and Critical Care Medicine, Graduate School of Biomedical and Health Sciences, Hiroshima University, Hiroshima, Japan; 9 Division of Clinical Laboratory and Infection Control, Yamagata University Hospital, Yamagata, Japan; 10 Biostatistics, Clinical and Translational Research Center, Keio University School of Medicine, Tokyo, Japan; 11 Faculty of Data Science, Yokohama City University School of Data Science, Yokohama, Japan; 12 Department of Pharmacy, St. Marianna University Hospital, Kanagawa, Japan; 13 Intensive Care Unit, Tohoku University Hospital, Miyagi, Japan; 14 Department of Anesthesiology and Critical Care Medicine, Jichi Medical University Saitama Medical Center, Saitama, Japan; 15 Department of Emergency and Critical Care Medicine, Okayama Saiseikai General Hospital, Okayama, Japan; 16 Intensive Care Unit, Mitsui Memorial Hospital, Tokyo, Japan; 17 Department of Anesthesiology and Intensive Care Medicine, International University of Health and Welfare, School of Medicine, Chiba, Japan; 18 Intensive Care Unit, Sakai city medical center, Osaka, Japan; 19 Emergency And Critical Care Medicine, Nerima Hikarigaoka Hospital, Tokyo, Japan; 20 Division of Critical Care Medicine, Saiseikai Utsunomiya Hospital, Tochigi, Japan; 21 Department of Emergency and Intensive Care Medicine, JA Hiroshima General Hospital, Hiroshima, Japan; 22 Division of Intensive Care Unit, Nagasaki University Hospital, Nagasaki, Japan; 23 Emergency Medical Center, Kagawa University Hospital, Kagawa, Japan; 24 Division of Emergency Medicine, Japanese Red Cross Kyoto Daiichi Hospital, Kyoto, Japan; 25 Intensive Care Unit, Okinawa Chubu Hospital, Okinawa, Japan; 26 Critical Care Center, Yamagata University Hospital, Yamagata, Japan; 27 Intensive Care Unit, Kochi Medical School Hospital, Kochi, Japan; 28 Intensive Care Unit, Shiroyama Hospital, Osaka, Japan; 29 Department of Acute Care and General Medicine, Saiseikai Kumamoto Hospital, Kumamoto, Japan; 30 Emergency Intensive Care Unit, Fujita Health University, Nagoya, Japan

**Keywords:** Catheter, Catheter-Related Infections, Critically ill patient, Epidemiology, Intensive care unit, Phlebitis

## Abstract

**Background:**

The lack of precise information on the epidemiology of peripheral intravascular catheter (PIVC)-related phlebitis and complications in critically ill patients results in the absence of appropriate preventive measures. Therefore, we aimed to describe the epidemiology of the use of PIVCs and the incidence/occurrence of phlebitis and complications in the intensive care unit (ICU).

**Methods:**

This prospective multicenter cohort study was conducted in 23 ICUs in Japan. All consecutive patients aged ≥ 18 years admitted to the ICU were enrolled. PIVCs inserted prior to ICU admission and those newly inserted after ICU admission were included in the analysis. Characteristics of the ICU, patients, and PIVCs were recorded. The primary and secondary outcomes were the occurrence and incidence rate of PIVC-related phlebitis and complications (catheter-related blood stream infection [CRBSI] and catheter failure) during the ICU stay.

**Results:**

We included 2741 patients and 7118 PIVCs, of which 48.2% were inserted in the ICU. PIVC-related phlebitis occurred in 7.5% (95% confidence interval [CI] 6.9–8.2%) of catheters (3.3 cases / 100 catheter-days) and 12.9% (95% CI 11.7–14.2%) of patients (6.3 cases / 100 catheter-days). Most PIVCs were removed immediately after diagnosis of phlebitis (71.9%). Grade 1 was the most common phlebitis (72.6%), while grade 4 was the least common (1.5%). The incidence rate of CRBSI was 0.8% (95% CI 0.4–1.2%). In cases of catheter failure, the proportion and incidence rate per 100 intravenous catheter-days of catheter failure were 21% (95% CI 20.0-21.9%) and 9.1 (95% CI 8.7–10.0), respectively.

**Conclusion:**

PIVC-related phlebitis and complications were common in critically ill patients. The results suggest the importance of preventing PIVC-related complications, even in critically ill patients.

**Trial registration:**

UMIN-CTR, the Japanese clinical trial registry (registration number: UMIN000028019, July 1, 2017).

**Supplementary Information:**

The online version contains supplementary material available at 10.1186/s40560-020-00518-4.

## Background

Peripheral intravascular catheters (PIVCs) are the most commonly used invasive medical devices in the intensive care unit (ICU), but complications associated with PIVCs, such as phlebitis, often occur [1]. Phlebitis causes pain, anxiety, and therapy interruption and can potentially lead to serious complications (e.g., skin necrosis, infective endocarditis) [2–4]. Other PIVC-related complications, such as accidental removal, occlusion, thrombosis, and PIVC-related infection, are also problematic [5–10].

The epidemiology of PIVCs and PIVC-related complications in general wards has been described in several studies. PIVCs are inserted in approximately 70% of hospitalized patients [11, 12]. The most common and important PIVC-related complication is phlebitis, which occurs with 7–44% of catheters [13–16]. The occurrence of PIVC-related complications, including phlebitis, accidental removal, occlusion, thrombosis, and catheter-related blood stream infection (CRBSI), is 20–50% [8, 17–19]. PIVC-related complications are also associated with the increase in mortality, morbidity, length of hospital stay, and hospital costs [20–24]. Several risk factors in terms of insertion sites, catheter types, dressing material types, administered drugs, and catheter-days have been identified [9, 10, 25–28]. However, the external validity of these findings to the ICU population is questionable.

The epidemiology of the use of PIVC and its complications, especially phlebitis in ICU patients, is largely unknown. A study found that PIVC-related complications occur in 30.6% of PIVCs in the ICU. However, further details (e.g., the occurrence of phlebitis) were not reported because that study was not focused solely on PIVCs [29]. To our knowledge, no study has described the occurrence of PIVC-related phlebitis and complications in the ICU. The lack of precise information on the epidemiology of phlebitis and complications in critically ill patients results in the absence of appropriate preventive measures. Therefore, we aimed to describe the epidemiology of the use of PIVCs and the incidence/occurrence of phlebitis and complications in the ICU.

## Methods

### Study design and setting

This prospective multicenter cohort study was conducted in 23 ICUs in Japan from January 1, 2018, to March 31, 2018. Prior to the study, we conducted a pilot study for 2 weeks in July 2017 in the ICU of Kameda Medical Center.

### Study pre-registration and ethics

The study was pre-registered at UMIN-CTR, the Japanese clinical trial registry (registration number: UMIN000028019). This study was approved by the institutional review board or medical ethics committee of each study institution. The need for informed consent was waived, and an opt-out recruitment method was employed.

### Study participants

**Fig. 1:**
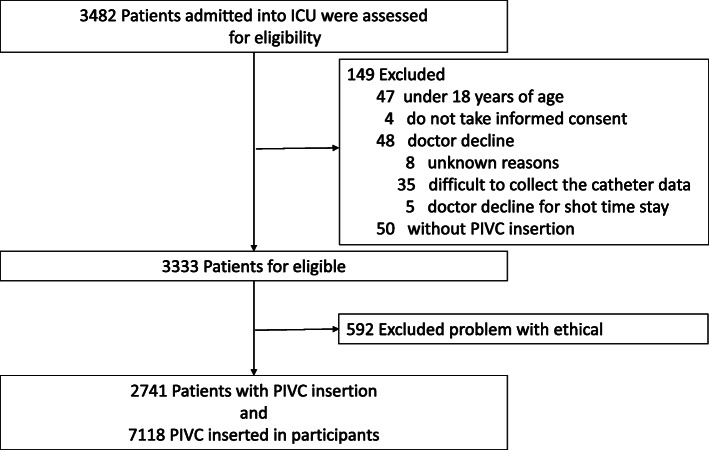
Patient flow chart

All consecutive patients aged ≥ 18 years admitted to the ICU during the study period were included. Patients who declined to participate in this study, had no PIVC during their ICU stay, and those who were selected for exclusion by the physicians (e.g., due to a short stay in the ICU or difficulty in collecting catheter data for various reasons) were excluded (Fig. 1).

### PIVCs

All PIVCs, those newly inserted into blood vessels after ICU admission and those inserted prior to ICU admission, were included. The following selections were left to the discretion of the physicians at each study institution: catheter type (e.g., material and catheter gauge), medical staff inserting PIVCs, insertion method (e.g., insertion site, antiseptic solution, use of ultrasound, and glove type), management method (e.g., type of dressing and timing of changing the dressing), and removal timing.

### Data collection

This study collected the following information: ICU characteristics, patient characteristics, PIVC characteristics, information on drugs administered via PIVC (e.g., drug concentration, administration rate, and administration duration), and outcomes. The study database was built using REDCap (Vanderbilt University Medical Center, Nashville, TN) data management tool. Data management was monitored via a data management center at Kameda Medical Center. To reduce information bias due to data input error, a data input test of a fictitious case was conducted in December 2017 before starting the main study, and each researcher was permitted to input data of the main study into REDCap after completing the input practice.

#### ICU characteristics

We distributed a key informant survey at each site to collect data on ICU characteristics, such as the number of beds in the ICU, provision of education on venous catheter management, presence of regular catheter replacement, presence of an established definition of phlebitis and CRBSI in the ICU, type of ICU patients (surgical, medical, or mixed), number of full-time equivalent ICU physicians per day, standardized drug administration measures in the ICU (standardized education *on* drug administration and combination contraindication tables), presence of a pharmacist in the ICU (e.g., full-time or daytime), and presence of nurses with specialized education on managing venous catheters at each institution (named IV nurse; no specific definition of specialized education or IV nurse was employed).

#### Patient characteristics

Data collected included patient demographics, medical history (Charlson comorbidity index [30], hypertension, and dyslipidemia), ICU admission route (emergency department, ward, or transfer from other hospital), disease type at ICU admission (scheduled operation, emergency operation, medical emergency), presence of sepsis at ICU admission (defined by sepsis-3 criteria [31]), length of ICU stay, and length of hospital stay. The following data were collected within the first 24 h after ICU admission: vital signs and laboratory data needed for calculation of acute physiology and chronic health evaluation (APACHE) II [32], simplified acute physiology score (SAPS) II [33], and Sequential Organ Failure Assessment (SOFA) score [34].

#### Catheter characteristics

The following variables were collected for all PIVCs, regardless of the insertion location: insertion date, removal date, insertion site, place of insertion and removal (in the ICU or outside the ICU), catheter material (description of trade name), catheter gauge, dressing method (gauze, non-sterile dressing, sterile dressing, or chlorhexidine-impregnated dressing), presence of any types of infections during catheterization, and types of complications and their date of occurrence (phlebitis, catheter occlusion, catheter-related infection, bacteremia, arterial puncture, hematoma, extravasation, nerve injury, tendon injury, skin necrosis, and compartment syndrome). In particular, when PIVCs were inserted in the ICU, additional data were collected. These data included the medical staff inserting the catheter, the number of punctures, skin antiseptics, types of gloves (non-sterile or sterile), and difficulty of insertion. In addition, with regard to the insertion site of the PIVC, the puncture site was described every 4 h (details are described in the “Study outcomes” section). Catheter information was recorded only during the patient’s ICU stay.

#### Drug characteristics

We gathered all information related to drugs administered through each PIVC, excluding catheter lock solutions such as heparin and saline. The information included drug composition, drug concentration, starting time of drug administration, time of changing the rate of drug administration, rate of drug administration, and stopping time of drug administration. These drug data were obtained from the electronic medical records and sent to the data management center. Drug information was recorded only during the ICU stay.

### Study outcomes

#### Primary outcomes

The primary outcome was occurrence of phlebitis during the ICU stay. Occurrence was reported as the proportion per total number of catheters and patients and the incidence rate per 100 catheter-days and 100 patient-days.

#### Definition of phlebitis and measurement

Phlebitis was defined using the Phlebitis Scale developed by the American Infusion Nurse Society (INS) [35] (e-Table 1, see Additional File 1). Catheter insertion sites were observed every 4 h by a bedside nurse for clinical signs relevant to phlebitis, namely, pain, erythema, edema, palpable venous cord, steak formation, and palpable venous cord > 1 in. (e-Table 2, see Additional File 1). These six variables were collected as binary variables. Phlebitis diagnosis was made simply based on the criteria composed of the six clinical signs by a blinded assessor at the main facility and was classified into four grades (e-Table 1, see Additional File 1). If patients could not assess their symptoms such as pain, well-trained nurses defined in each study institution judged the grade of pain. To reduce information bias, pilot trainings were conducted for the accuracy of phlebitis diagnosis; statistical analysis was not performed. Furthermore, the accuracy of phlebitis diagnosis was monitored during the study period at the central institution by a well-trained researcher, who could properly diagnose phlebitis defined using the Phlebitis Scale developed by the INS. This was performed by confirming the accuracy of the information on catheter insertion site from the phlebitis images, which were sent to the data management center from each study institution for the first month after starting data collection.

Further information on the primary outcomes, which included the characteristics of phlebitis, such as occurrence date, phlebitis grade, and the frequency of each element of phlebitis definition, and information on the following subgroups were also described: location where the PIVC was inserted (before or after ICU admission; before ICU divided into two categories; emergency room (ER), and others including operation room (OR), general wards, and outpatients) and admission type to ICU (elective surgery, emergency surgery, and medical emergency).

#### Secondary outcomes

The secondary outcomes included (1) the occurrence of CRBSIs according to the proportion and the incidence rate per 1000 catheter-days and per 100 patient-days, defined by the Infectious Disease Society of America [36] or Centers for Disease Control and Prevention [37], which are the commonly used definitions of CRBSI of central venous catheters (CVCs) (e-Table 3, see Additional File 1), and (2) the occurrence of PIVC failure according to the proportion and the incidence rate per 100 catheter-days and per 100 patient-days. PIVC failure was defined as a composite outcome including the following 15 complications leading to catheter removal: phlebitis, obstruction, CRBSI, arterial puncture, hematoma, extravasation, nerve injury, tendon injury, extreme compartment, accidental removal, pain, redness, inappropriate insertion site (e.g., close to other catheters, or disturbance of medical restraint), effusion around insertion site, and others. Definitions of each complication other than phlebitis and CRBSI were left to each study institution.

### Statistical methods

The target sample size in this study was 1825 catheters and was calculated as follows: the phlebitis occurrence was 5% of PIVCs according to our pilot study (unpublished), which was reported at the 44th annual meeting of the Society of Japanese Intensive Care Medicine, and the estimated 95% confidence interval (CI) was 3–6%.

Patient characteristics, catheter characteristics, and primary and secondary outcomes were presented as mean with standard deviation (SD) or median with interquartile range (IQR) for continuous variables and percentages for categorical variables. Proportion, time to event, and incidence rate of outcomes were presented as each effect size with two-tailed 95% CIs calculated using the Wilson score interval and Poisson distribution. Analyses were performed using JMP V.10.0 and SAS version 9.4 (SAS Inc, Cary, NC).

## Results

### Participating patients

A total of 3482 patients from 23 ICUs were reviewed for eligibility within the study period, of which 149 were not eligible, with 50 not having a PIVC (Fig. 1). Of the 3333 patients remaining, 592 from 4 institutions were excluded due to ethical issues (non-anonymized data). Finally, 2741 patients and 7118 PIVCs were analyzed.

### Patient characteristics

Table 1 shows the characteristics of patients with PIVCs inserted at ICU admission. The mean ± SD age of the study population was 67.3 ± 15.5 years. In addition, 60.6% of all patients were admitted to the ICU from the OR, followed by 27.4% from the ER. The most common type of ICU admission was postoperative, comprising 60.6% of participants (46.5% for elective operation and 14.1% for emergency operation). The mean ± SD severity score of the included patients was 16.2 ± 7.9 for APACHE II and 5.2 ± 3.6 for SOFA score. Sepsis accounted for 11.6% of cases.

**Table 1:** Patient characteristics with PIVC inserted at ICU admission

Variables	Total *n* = 2741
Age, mean (SD), years	67.3 (15.5)
Gender, male (*n*, %)	1682 (61.4%)
Body height, mean (SD), cm ^a^	161 (9.8)
Body weight, mean (SD), kg ^b^	59.1 (14.1)
BMI, mean (SD) ^a^	22.8 (4.3)
APACHEII, mean (SD) ^c^	16.2 (7.9)
SAPSII, mean (SD) ^c^	35.8 (19.7)
SOFA, mean (SD) ^c^	5.2 (3.6)
Charlson comorbidity index, mean (SD)	4.4 (2.6)
ICU admission from (*n*, %)	
Operation room	1661 (60.6%)
Emergency room	750 (27.4%)
General ward	249 (9.1%)
Outpatients	17 (0.6%)
Transfer from other hospital	64 (2.3%)
Type of admission to ICU (*n*, %)	
Elective operation	1275 (46.5%)
Emergency operation	386 (14.1%)
Medical emergency	1080 (39.4%)
ICU admission category (*n*, %)	
Cardiology	919 (33.5%)
Pulmonary	363 (13.2%)
Gastrointestinal	505 (18.4%)
Neurology	315 (11.5%)
Trauma	98 (3.6%)
Urology	74 (2.7%)
Gynecology	59 (2.2%)
Skin/tissue	53 (1.9%)
Others	355 (13.0%)
Sepsis at ICU admission (*n*, %) ^d^	
Sepsis	319 (11.6%)
Septic shock	188 (6.9%)
Mechanical ventilation within 24 h after admission to ICU (*n*, %) ^e^	1713 (64.2%)
Non-invasive ventilation	140 (5.3%)
Invasive ventilation	814 (30.5%)

*APACHE* acute physiology and chronic health evaluation, *BMI* body mass index, *ICU* intensive care unit, *SAPS* simplified acute physiology score, *PIVC* peripheral intravenous catheter, *SD* standard deviation, *SOFA* sequential organ failure assessment

^a^Missing data: *n* = 3

^b^Missing data: *n* = 2

^c^Missing data: *n* = 171

^d^Missing data: *n* = 1

^e^Missing data: *n* = 74

### PIVC characteristics

Table 2 presents the characteristics of the 7118 PIVCs. Catheters inserted after ICU admission comprised 48.2% of the PIVCs. The most frequently used site of insertion was the forearm (56.2%), followed by the back of the hand (21.3%). The most commonly inserted catheter gauge was 22G (49.0%), followed by 20G (35.2%). The rate of infections during catheterization was 17.8%. Of the 7118 PIVCs, 3705 (52.2%) were removed prior to ICU discharge, with a median PIVC insertion duration of 36 h and an IQR of 19.0–71.1 h.

**Table 2:** All PIVC characteristics during insertion

Variables	Total *n* = 7118
Location of insertion	
ICU	3429/7115 (48.2%)
ER	1310/7115 (18.4%)
Others (ward, OR, outpatients)	2376/7115 (33.4%)
Inserted site (*n*,%)	
Upper arm	473/7097 (6.7%)
Forearm	3986/7097 (56.2%)
Elbow	355/7097 (5.0%)
Wrist	286/7097 (4.0%)
Hand	1514/7097 (21.3%)
Lower leg	283/7097 (4.0%)
Dorsal foot	169/7097 (2.4%)
Others	31/7097 (0.4%)
Catheter material (*n*,%)	
PEU-Vialon^a^	2120/7118 (29.8%)
Polyurethane	1968/7118 (27.7%)
Polyethylene	0/7118 (0%)
Tetrafluoroethylene	2695/7118 (37.9%)
Others	335/7118 (4.8%)
Catheter gauge (*n*,%)	
14G	10/7009 (0.1%)
16G	386/7009 (5.5%)
18G	595/7009 (8.5%)
20G	2464/7009 (35.2%)
22G	3432/7009 (49.0%)
23G	1/7009 (0.01%)
24G	121/7009 (1.7%)
Dressing (*n*,%)	
Chlorhexidine-impregnated dressing	1/7043 (0.01%)
Sterile polyurethane dressing	6844/7043 (97.2%)
Non-sterile polyurethane dressing	85/7043 (1.2%)
Gauze dressing	2/7043 (0.03%)
Tape dressing	111/7043 (1.6%)
Any infection during catheterization (*n*,%)	1267 (17.8%)
Duration of catheterization, median (IQR), hours	36.0 (19.0-71.0)
Removal in ICU (*n*, %)	3705/7103 (52.2%)

*ICU* intensive care unit, *IQR* interquartile range, *PIVC* peripheral intravenous catheter, *ER* emergency room, *OR* operation room, ^a^*PEU-Vialon* polyetherurethane without leachable additives

Table 3 shows the characteristics of PIVCs inserted in the ICU. Out of the 3429 PIVCs inserted in the ICU, 21.8–24.4% were excluded from the detailed description shown in Table 3 because data were missing for unknown reasons. The rate of ultrasound utilization was 2.2%, and most PIVCs were successfully inserted with a single puncture (80.9%), but in some cases, more than four punctures were required (1.2%). In most cases (77.2%), the operator felt that the insertion was relatively easy, but 22.8% of the catheters were difficult to insert.

**Table 3:** PIVC characteristics inserted in ICU

Variables	Total *n* = 3429
Catheter inserted by (*n*,%)	
Doctor	287/2681 (10.7%)
Nurse	2393/2681 (89.3%)
Medical technologist	1/2681 (0.04%)
Antiseptic solution before catheterization (*n*,%)	
None	8/2665 (0.3%)
Alcohol	2599/2665 (97.5%)
0.2% chlorhexidine alcohol	21/2665 (0.8%)
0.5% chlorhexidine alcohol	15/2665 (0.6%)
1.0% chlorhexidine alcohol	17/2665 (0.7%)
10% povidone iodine	2/2665 (0.1%)
Other	3/2665 (0.1%)
Use of ultrasonography (*n*,%)	58/2636 (2.2%)
Number of trials for insertion (*n*,%)	
1	2119/2619 (80.9%)
2	313/2619 (12.0%)
3	130/2619 (5.0%)
4	26/2619 (1.0%)
5	15/2619 (0.6%)
≧ 6	16/2619 (0.6%)
Difficulty of insertion (*n*,%)	
Easy	1232/2594 (47.5%)
Slightly easy	772/2594 (29.8%)
Slightly difficult	456/2594 (17.6%)
Difficult	134/2594 (5.2%)
Glove (*n*,%)	
Sterile	19/2630 (0.7%)
Non-sterile	2496/2630 (94.9%)
Nothing	115/2630 (4.4%)

*ICU* intensive care unit, *PIVC* peripheral intravenous catheter

### Incidence and characteristics of phlebitis and catheter failure

**Fig. 2:**
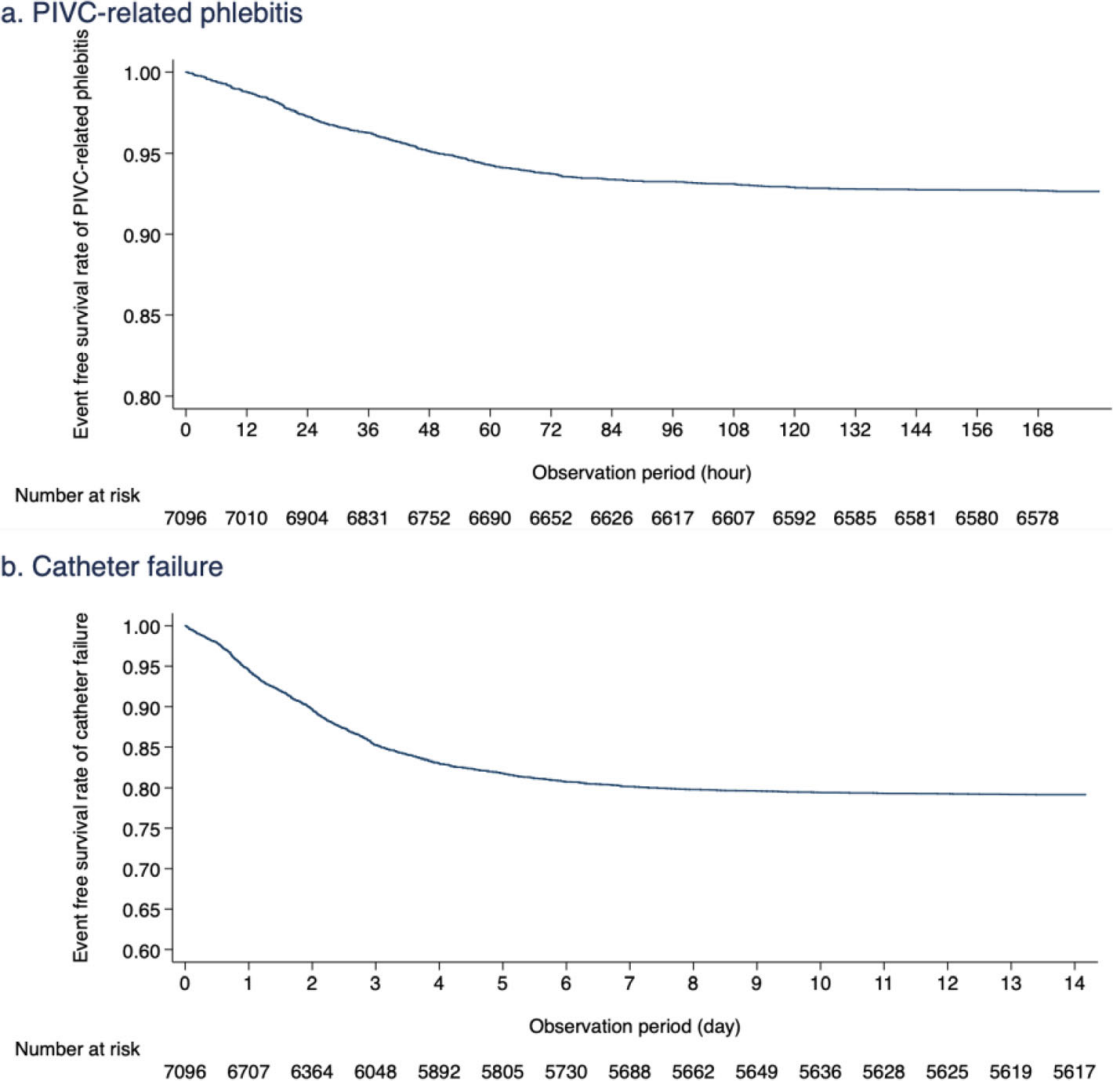
Event-free survival rate of phlebitis and catheter failure. **a** PIVC-related phlebitis. **b** Catheter failure

**Fig. 3:**
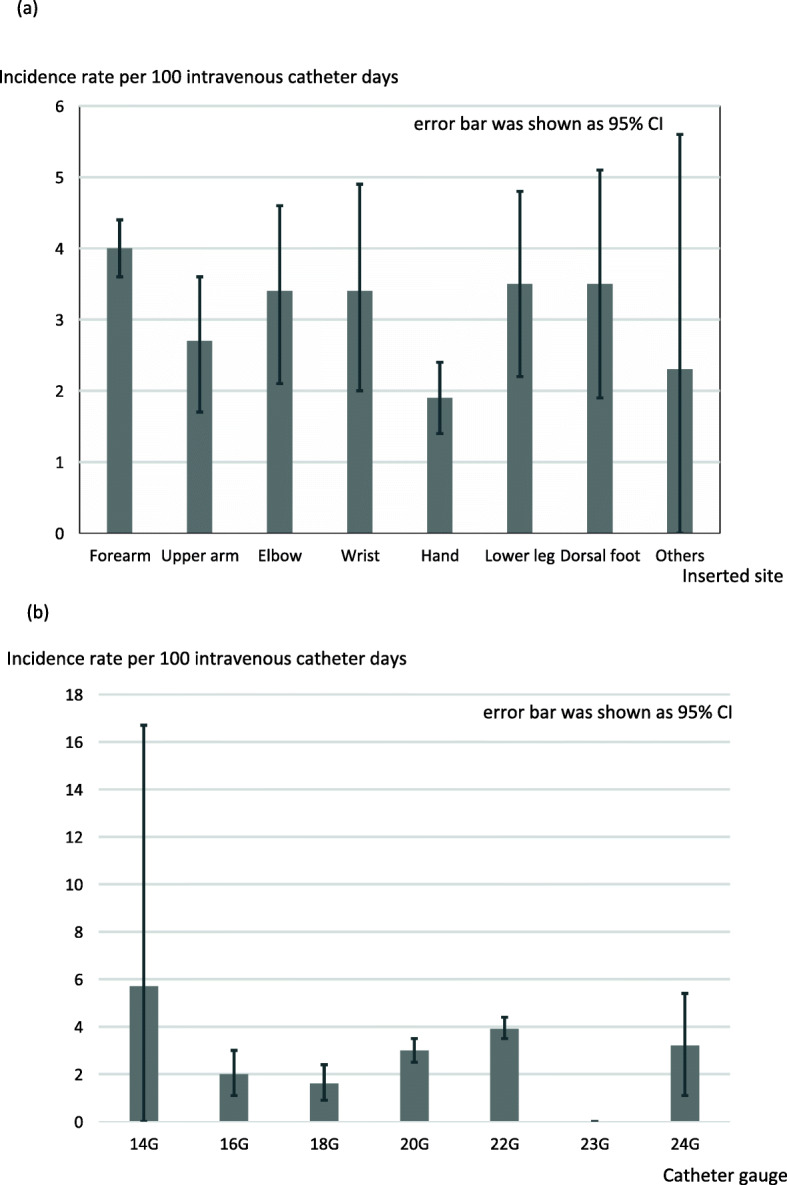
Incidence rate per 100 intravenous catheter-days of phlebitis according to each inserted site and catheter gauge. **a** Inserted site. **b** Catheter gauge

Phlebitis as a primary outcome occurred in 535 of 7118 PIVCs (7.5%; 95% CI 6.9–8.2%, Table 4). The incidence rate of phlebitis per 100 catheter insertion days was 3.3 (95% CI 3.0–3.6). The proportion and incidence rate of phlebitis per patient and the 95% CI, not per catheter, were 12.9 (95% CI 11.7–14.2) and 6.3 (95% CI 5.6–6.9), respectively (e-Table 4, see Additional File 1). The event-free survival rate of phlebitis is shown in Fig. 2a as a Kaplan-Meier curve. Table 5 shows each element of phlebitis, phlebitis grade, and number of phlebitis findings confirmed prior to catheter removal. Most PIVCs were removed after one or two diagnoses of phlebitis (83.6%); however, 7.6% of PIVCs were not removed even though phlebitis was observed more than four times. With regard to the grade of phlebitis observed, grade 1 was the most common (72.6%). The proportion of the severest grade of phlebitis (grade 4) was 1.5%. The proportion and incidence rate of phlebitis per insertion site and catheter gauge were shown in Fig. 3 and e-Table 5 (see Additional File 1).

**Table 4:** Primary and Secondary outcomes per catheter

Primary outcome		
Phlebitis per catheter	Number of catheters/total PIVCs	535/7118
	Proportion, % (95% CI)	7.5 (6.9–8.2)
	Time to phlebitis^a^, median (IQR), hours	36.3 (17.8–58.6)
	Incidence rate per 100 intravenous catheter-days (95% CI)	3.3 (3.0–3.6)
Secondary outcomes		
CRBSI	Number of catheters/total PIVCs	3/7025
	Proportion, % (95% CI)	0.04 (0.02–0.13)
	Time to removal^b^, median (IQR), hours	481.5 (12.8–558.3)
	Incidence rate per 1000 intravenous catheter-days (95% CI)	0.18 (0–0.39)
Catheter failure	Number of catheters/total PIVCs	1492/7118
	Proportion, % (95% CI)	21.0 (20.0–21.9)
	Time to removal^b^, median (IQR), hours	48.3 (23.3–81.9)
	Incidence rate per 100 intravenous catheter-days (95% CI)	9.1 (8.7–10.0)

*CI* confidence interval, *CRBSI* catheter-related blood stream infection, *ICU* intensive care unit, *IQR* interquartile range, *PIVC* peripheral intravenous catheter

^a^Time to phlebitis means the duration between insertion of the catheter and detection of phlebitis

^b^Time to removal means the duration between insertion and removal of the catheter

**Table 5:** Characteristics of phlebitis

Variables		Total *n* = 535
Clinical sign of insertion site		
Pain/total PIVCs		147/382 (38.5%)
Erythema (*n*, %)		512/535 (95.7%)
Edema (*n*, %)		157/514 (30.6%)
Streak formation (*n*, %)		95/516 (18.4%)
Palpable venous cord (*n*, %)		24/510 (4.7%)
Palpable venous cord > 1 in. (*n*, %)		17/507 (3.4%)
No. of times phlebitis observed before removal (*n*, %)		
1		388/535 (71.9%)
2		63/535 (11.7%)
3		28/535 (5.2%)
4		20/535 (3.7%)
5		29/535 (5.4%)
> 5		12//535 (2.2%)
Maximal grade of phlebitis		
1	(*n*, %)	387/535 (72.3%)
	Removed in ICU	372/387 (96.1%)
	Time to removal^a^, median (IQR), hours	36.9 (18.3–61.1)
2	(*n*, %)	130/535 (24.3%)
	Removed in ICU	127/130 (97.7%)
	Time to removal^a^, median (IQR), hours	31.5 (16.5–54.1)
3	(*n*, %)	10/535 (1.9%)
	Removed in ICU	10/10 (100%)
	Time to removal^a^, median (IQR), hours	21.9 (17.1–60.1)
4	(*n*, %)	8/535 (1.5%)
	Removed in ICU	8/8 (100%)
	Time to removal^a^, median (IQR), hours	38.7 (35.6–47.9)

*CI* confidence interval, *CRBSI* catheter-related blood stream infection, *ICU* intensive care unit. *IQR* interquartile range. *PIVC* peripheral intravenous catheter

^a^Time to removal means the duration between insertion and removal of the catheter

Table 4 shows the results of the secondary outcomes analysis. CRBSI occurred in 3 catheters (0.04%, 95% CI 0.02–0.13%) with an incidence rate of 0.18 (95% CI 0–0.39). The median time-to-onset of CRBSI was 481.5 h (95% CI 12.8–558.3). The three cases of CRBSI were caused by *methicillin-resistant Staphylococcus aureus*, *Candida albicans*, and *Corynebacterium spp*. None of the cases of CRBSI resulted in complications such as infective endocarditis or infectious arthritis. With regard to catheter failure, the proportion and incidence rate per 100 intravenous catheter-days were 21% (95% CI 20.0–21.9%) and 9.1 (95% CI 8.7–10.0), respectively. The event-free survival rate of phlebitis is shown in Fig. 2b as a Kaplan-Meier curve. Incidence rates for each component of catheter failure are shown in e-Table 6 (see Additional File 1). The most frequent PIVC failure was extravasation (7.7%), followed by phlebitis (7.5%).

### PIVC characteristics of subgroups

Regarding the subgroup descriptions, e-Table 7 shows the differences in the characteristics of the PIVCs and the incidence of phlebitis depending on the location of PIVC insertion (see Additional File 1). The incidence rate of phlebitis per 100 intravenous catheter-days at each insertion location was 7.8 (95% CI 6.9–8.7) for PIVCs inserted in the ICU, 3.9 (95% CI 3.2–4.6) for PIVCs inserted in the ER, and 1.1 (95% CI 0.9–1.3) for PIVCs inserted in other locations. Similar differences in the occurrence of phlebitis were observed among admission-type subgroups (e-Table 8, see Additional File 1).

## Discussion

This study presented the largest and most detailed description of the epidemiology of PIVCs and PIVC-related complications in critically ill patients. PIVC-related phlebitis occurred in 7.5% of catheters (3.3 cases/100 catheter-days) and 12.9% of patients (6.3 cases/100 catheter-days). These rates differed depending on insertion location and type of admission to ICU.

A study described the epidemiology of PIVC-related complications in critically ill patients [29]. In total, 2214 catheters (e.g., PIVCs, CVC, dialysis catheter, pulmonary arterial catheter, and arterial catheter) inserted in the ICUs, of which 873 were PIVCs, were included in that study. The occurrence and incidence rate of complications with PIVCs, which included superficial phlebitis and extravasation, were 5.2% and 24.7 cases/1000 catheter-days, respectively, which were lower than those of our study. These differences might be attributed to the presence of strict protocols and education on catheter management. That previous study [29] involved well-trained staff members who were aware of institutional protocols for catheter insertion, maintenance, and surveillance, following the French Haute Autorité de Santé Checklist and international guidelines for preventing catheter-related infections. Our study did not prepare a PIVC management protocol and did not educate the staff on PIVC management other than the prescribed observation of inserted PIVCs every 4 h. However, phlebitis incidence reportedly decreases when well-trained nurses managed the entire process of PIVC management from insertion to removal [38]. This randomized prospective controlled study compared PIVCs inserted and maintained by the IV team to those inserted and maintained by the medical house staff for the prevention of catheter complications including phlebitis. The rate of phlebitis decreased from 1.5 to 0.1% for insertion and maintenance by the IV team. However, only 4 of the 19 institutions in our study had IV nurses. The lack of a strict protocol and education on PIVC management, as well as well-trained IV nurses, may have led to the increased occurrence of phlebitis in our study. However, the present results may reflect the real occurrence of phlebitis in critically ill patients because quite a few institutions and ICUs, not only in Japan but also in other countries, do not have well-trained IV nurses.

Compared to the studies on critically ill patients, many studies have reported the incidence of phlebitis in non-critically ill patients, and the occurrence has been widely reported as 7–44% depending on the patients [13–16]. The wide range of phlebitis occurrence may be due to the heterogeneity of the included patients and catheters in the studies, but numerous studies have reported that phlebitis occurrence in non-critically ill patients is assumed to be around 10–20%. The incidence of phlebitis in critically ill patients was slightly lower than that in non-critically ill patients. There are some possible reasons for this difference. First, the insertion rate of CVCs other than peripheral venous catheters is usually high in critically ill patients, and drugs that are risk factors for phlebitis development may be administered via CVCs. The patients who have a high risk of phlebitis, such as difficulty of insertion and high-risk drugs, tend to have CVCs inserted instead of PIVCs, which may result in the lower risk of phlebitis in critically ill patients. Second, the frequency of observation of the catheter insertion site in critically ill patients will be higher than that in non-critically ill patients. Therefore, the PIVCs in critically ill patients may receive early intervention at an earlier stage of phlebitis that does not meet the criteria for phlebitis, such as only redness at the insertion site (observation bias). Finally, phlebitis with INS criteria is not diagnosed only with redness at the catheter insertion site. Therefore, the incidence may be low when intervention, such as discontinuation or change of drug, is performed at the stage of redness alone. Although the incidence of phlebitis in critically ill patients was slightly lower than that in non-critically ill patients, this does not indicate that PIVCs inserted in critically ill patients are safer than those in non-critically ill patients. When PIVCs inserted in critically ill patients are used inappropriately, the complications may increase. A study reported in 2013, which compared CVCs and PIVCs as the initially used catheter in the ICU, showed that the risk of complications increased when PIVCs were used inappropriately [39]. In that study, the protocol allowed the use of noradrenaline at up to 2 mg/h through PIVCs. This amount of noradrenaline is extremely high for administration via PIVCs, which may lead to increased PIVC-related complications.

### Limitations

This study has several limitations. First, selection bias may exist in this study. Four of 23 study institutions were excluded from this study because of ethics violation involving sending of non-anonymized patient data to the data management center. As a result, 592 (17.8%) patients were excluded. If these excluded data were missing not at random, the selection bias affects the internal validity of this study. However, since all data from the four study institutions were excluded, and the data from the other 19 study institutions were consecutively collected, selection bias is considered to be minimal. Second, the data collected were limited to information on PIVC insertion and drugs administered during ICU admission. Detailed information on PIVC insertion before ICU admission, information on catheter insertion site after ICU discharge, and the presence or absence of complications, such as phlebitis, were not examined (derived before ICU discharge, only complications were considered). Information regarding drugs administered via catheters before ICU admission and after ICU discharge was not included. Therefore, phlebitis incidence may be underestimated. Third, the final diagnosis of secondary outcomes, including CRBSI, was left to each institution, which may have led to misclassification of secondary outcomes. For example, the definition of CRBSI requires exclusion of other infections, including CRBSI by the CVC, but there is no confirmation as to how these were excluded. Therefore, the accuracy of the diagnosis is unclear. Fourth, the risk factors for PIVC-related phlebitis could not be examined in the present study. Although it is very important to examine risk factors for PIVC-related phlebitis for the clinical application of the results of this study, information on peripheral venous catheters in critically ill patients is not available. Because of the lack of epidemiological information, represented by the frequency of complications, our primary goal was to first present the epidemiology of the disease. Finally, the diagnostic criteria for phlebitis used require pain assessment at the catheter insertion site, but pain assessment in critically ill patients is insufficient due to impaired consciousness and sedation. Thus, phlebitis misdiagnosis is possible. Developing new diagnostic criteria for phlebitis that can be used in even severely ill patients may be necessary.

### Implications

This study could provide epidemiological data on phlebitis and complications in critically ill patients. The incidence of PIVC-related complications may differ between critically ill patients and non-critically ill patients. The present results could lay an important foundation for future studies on PIVC-related complications in critically ill patients, such as the identification of risk factors for phlebitis and complications and the development of models for the prediction of phlebitis.

## Conclusions

PIVC-related phlebitis and complications were common in critically ill patients. The results suggest the importance of preventing PIVC-related complications even in critically ill patients. This study puts into perspective phlebitis and complications in critically ill patients, such as prevention methods for these complications.

## Supplementary Information

**Additional file 1:** **e-Table 1**. The definition of phlebitis (INS). **e-Table 2**. The definition of each element of the INS’s phlebitis definition. **e-Table 3**. Definition of catheter-related blood stream infection. (A) A definitive diagnosis of CRBSI suggested by the CDC. (B) A definitive diagnosis of CRBSI suggested by the IDSA. **e-Table 4.** Primary outcome per patient. **e-Table 5**. Primary outcome according to each inserted site and catheter gauge. **e-Table 6**. Each component of PIVC failure per catheter. **e-Table 7**. Characteristics of PIVCs and outcomes according to insertion location. **e-Table 8**. Characteristics of PIVCs and outcomes according to insertion location admission type.

## Data Availability

The datasets generated during and/or analyzed during the current study are not publicly available due to post hoc analyses by the co-authors but are available from the corresponding author on reasonable request.

## Abbreviations

APACHE: Acute physiology and chronic health evaluation
CI: Confidence interval
CRBSI: Catheter-related blood stream infection
CVC: Central venous catheter
ER: Emergency room
ICU: Intensive care unit
INS: Infusion Nurse Society
IQR: Interquartile range
OR: Operation room
PIVC: Peripheral intravascular catheters
SAPS: Simplified acute physiology score
SD: Standard deviation
SOFA: Sequential Organ Failure Assessment
