# Supplementary material for: Occurrence and incidence rate of peripheral intravascular catheter-related phlebitis and complications in critically ill patients: a prospective cohort study (AMOR-VENUS study)
Source: J Intensive Care. 2021 Jan 6;9:3. doi: 10.1186/s40560-020-00518-4 (PMC7789473; doi:10.1186/s40560-020-00518-4)
Supplement: Supplementary file 1 — Additional file 1: e-Table 1. The definition of phlebitis (INS). e-Table 2. The definition of each element of the INS’s phlebitis definition. e-Table 3. Definition of catheter-related blood stream infection. (A) A definitive diagnosis of CRBSI suggested by the CDC. (B) A definitive diagnosis of CRBSI suggested by the IDSA. e-Table 4. Primary outcome per patient. e-Table 5. Primary outcome according to each inserted site and catheter gauge. e-Table 6. Each component of PIVC failure per catheter. e-Table 7. Characteristics of PIVCs and outcomes according to insertion location. e-Table 8. Characteristics of PIVCs and outcomes according to insertion location admission type. [file 40560_2020_518_MOESM1_ESM.docx]

**Additional File 1**

**e-Table 1 The definition of phlebitis (INS)**

| Grade | Criteria |
| --- | --- |
| 0 | No clinical symptoms |
| 1 | Erythema at access site with or without pain |
| 2 | Pain at access site with erythema and/or edema |
| 3 | Pain at access site with erythema and/or edema, streak formation, palpable venous cord |
| 4 | Pain at access site with erythema and/or edema, or palpable venous cord > 1 inch, purulent drainage |

INS, infusion nurse society

**e-Table 2 The definition of each element of INS phlebitis definition**

| Element | definition |
| --- | --- |
| Pain | Pain around PIVC insertion site |
| Erythema | Redness around PIVC insertion site |
| Edema | Swelling around PIVC insertion site |
| Streak formation | Redness along blood vessels at PIVC insertion site |
| Palpable venous cord | Induration along blood vessels at PIVC insertion site |

INS, infusion nurse society; PIVC, peripheral intravenous catheter

**e-Table 3 Definition of Catheter-related blood stream infection**

1. A definitive diagnosis of CRBSI suggested by CDC

| Criteria 1 | The patient had a recognized pathogen cultured from ≥ 1 blood culture (the term ”recognized pathogen” does not include organisms considered as common skin contaminants, i.e., those that can be cultured from ≥2 blood cultures drawn on separate occasions). |
| --- | --- |
| Criteria 2 | The patient has one or more of the following signs/symptoms: fever ≥38.0°C, chills, or hypotension, unrelated to infection at any other site. |
| Criteria 3 | The same organism is grown from ≥1 percutaneous blood culture as well as the catheter-tip culture. |

CRBSI, Catheter-Related Bloodstream Infection; CDC, Center for Disease Control and Prevention

(B) A definitive diagnosis of CRBSI suggested by IDSA

| definition 1 | | The same organism grow from at least 1 percutaneous blood culture and from a culture of the catheter tip. |
| --- | --- | --- |
| definition 2 | | Two blood samples be drawn (one from a catheter hub and the other from a peripheral vein) that, when  cultured, meet CRBSI criteria for quantitative blood cultures* or DTP**. |
|  | |  |
| definition 3 | | Two quantitative blood cultures of samples obtained through 2 catheter lumens in which the colony count for  the blood sample drawn through one lumen is at least 3-fold greater than the colony count for the blood sample obtained from the second lumen should be considered to indicate possible CRBSI. |
|  | |  |
| * | For quantitative blood cultures, a colony count of microbes grown from blood obtained through the catheter hub that is at least 3-fold greater than the colony count from blood obtained from a peripheral vein best defines CRBSI. | |
|  |  |  |
|  |  | |
| ** | For DTP, growth of microbes from a blood sample drawn from a catheter hub at least 2 h before microbial growth is detected in a blood sample obtained from a peripheral vein best defines CRBSI. | |

CRBSI, Catheter-Related Bloodstream Infection; DTP, differential time to positivity; IDSA, the Infectious Diseases Society of America

**e-Table 4 Primary outcome per patient**

| Phlebitis per patient | Number of patients/total patients | 353/2741 |
| --- | --- | --- |
|  | Proportion, % (95% CI) | 12.9 (11.7-14.2) |
|  | Time to phlebitis*, median (IQR), hours | 27.1 (13.2-50.7) |
|  | Incidence rate per 100 intravenous catheter days (95% CI) | 6.3 (5.6-6.9) |

CI, confidence interval; CRBSI, catheter-related blood stream infection; ICU, intensive care unit; IQR, interquartile range; PIVC, peripheral intravenous catheter

*time to phlebitis means the duration between insertion of the catheter and detection of phlebitis.

**e-Table 5 Primary outcome according to each inserted site and catheter gauge**

| Inserted site | Forearm | Upper arm | Elbow | Wrist | Hand | Lower leg | Dorsal foot | Others |
| --- | --- | --- | --- | --- | --- | --- | --- | --- |
| Number of catheters/total PIVCs | 344/3,986 | 29/473 | 28/355 | 21/286 | 62/1,514 | 28/283 | 19/169 | 2/31 |
| Proportion, % (95% CI) | 8.6  (7.8-9.5) | 6.1  (4.3-8.7) | 7.9  (5.5-11.2) | 7.3  (4.9-11.0) | 4.1  (3.2-5.2) | 9.9  (6.9-13.9) | 11.2  (7.3-16.9) | 6.5  (1.8-20.7) |
| Time to phlebitis*, median (IQR), hours | 37.0  (17.5-56.7) | 44.3  (19.1-67.2) | 22.8  (17.9-50.9) | 20.0  (8.3-79.6) | 39.8  (19.2-78.3) | 30.6  (16.4-53.1) | 44.7  (26.5-68.7) | 31.7  (26.6-36.8) |
| Incidence rate per 100 intravenous catheter days (95% CI) | 4.0  (3.6-4.4) | 2.7  (1.7-3.6) | 3.4  (2.1-4.6) | 3.4  (2.0-4.9) | 1.9  (1.4-2.4) | 3.5  (2.2-4.8) | 3.5  (1.9-5.1) | 2.3  (0-5.6) |
|  |  |  |  |  |  |  |  |  |
| Catheter gauge | 14G | 16G | 18G | 20G | 22G | 23G | 24G |  |
| Number of catheters/total PIVCs | 1/10 | 18/386 | 17/595 | 159/2,464 | 320/3,432 | 0/1 | 9/121 |  |
| Proportion, % (95% CI) | 10.0  (1.8-40.4) | 4.7  (3.0-7.3) | 2.9  (1.8-4.5) | 6.5  (5.6-7.5) | 9.3  (8.4-10.3) | 0  (0-0) | 7.4  (4.0-13.5) |  |
| Time to phlebitis*, median (IQR), hours | 12.5  (12.5-12.5) | 66.9  (23.5-108.6) | 27.9  (10.2-71.0) | 32.4  (17.5-58.0) | 37.2  (18.0-56.7) | - | 25.0  (13.9-71.6) |  |
| Incidence rate per 100 intravenous catheter days (95% CI) | 5.7  (0-16.7) | 2.0  (1.1-3.0) | 1.6  (0.9-2.4) | 3.0  (2.5-3.5) | 3.9  (3.5-4.4) | 0  (0-0) | 3.2  (1.1-5.4) |  |

CI, confidence interval; IQR, interquartile range; PIVC, peripheral intravenous catheter

*time to phlebitis means the duration between insertion of the catheter and detection of phlebitis.

**e-Table 6 Each component of PIVC failure per catheter**

| Complication |  |  |
| --- | --- | --- |
| Catheter failure | Number of catheters/total PIVCs | 1,492/7,118 |
|  | Proportion, % (95% CI) | 21.0 (20.0-21.9) |
|  | Time to removal*, median (IQR), hours | 48.3 (23.3-81.9) |
|  | Incidence rate per 100 intravenous catheter days (95% CI) | 9.1 (8.7-10.0) |
| Phlebitis | Number of catheters/total PIVCs | 535/7,118 |
|  | Proportion, % (95% CI) | 7.5 (6.9-8.2) |
|  | Time to phlebitis**, median (IQR), hours | 36.3 (17.8-58.6) |
|  | Incidence rate per 100 intravenous catheter days (95% CI) | 3.3 (3.0-3.6) |
| Obstruction | Number of catheters/total PIVCs | 419/7,118 |
|  | Proportion, % (95% CI) | 5.9 (5.4-6.5) |
|  | Time to removal*, median (IQR), hours | 49.9 (23.8-84.9) |
|  | Incidence rate per 100 intravenous catheter days (95% CI) | 2.6 (2.3-2.8) |
| CRBSI | Number of catheters/total PIVCs | 3/7,025 |
|  | Proportion, % (95% CI) | 0.04 (0.02-0.13) |
|  | Time to removal*, median (IQR), hours | 481.5 (12.8-558.3) |
|  | Incidence rate per 1,000 intravenous catheter days (95% CI) | 0.18 (0-0.39) |
| Arterial puncture | Number of catheters/total PIVCs | 1/7,118 |
|  | Proportion, % (95% CI) | 0.01 (0.003-0.008) |
|  | Time to removal*, median (IQR), hours | 34.5 (34.5-34.5) |
|  | Incidence rate per 1,000 intravenous catheter days (95% CI) | 0.06 (0-0.18) |
| Hematoma | Number of catheters/total PIVCs | 57/7,118 |
|  | Percentage, % (95% CI) | 0.8 (0.6-1.0) |
|  | Time to removal*, median (IQR), hours | 43.0 (24.8-77.8) |
|  | Incidence rate per 1,000 intravenous catheter days (95% CI) | 3.5 (2.6-4.4) |
| Extravasation | Number of catheters/total PIVCs | 547/7,118 |
|  | Proportion, % (95% CI) | 7.7 (7.1-8.3) |
|  | Time to removal*, median (IQR), hours | 47.4 (21.8-75.0) |
|  | Incidence rate per 100 intravenous catheter days (95% CI) | 3.4 (3.1-3.6) |
| Nerve injury | Number of catheters/total PIVCs | 3/7,118 |
|  | Proportion, % (95% CI) | 0.04 (0.01-0.1) |
|  | Time to removal*, median (IQR), hours | 18.0 (17.8-19.4) |
|  | Nerve injury per 1,000 intravenous catheter days (95% CI) | 0.18 (0-0.39) |
| Tendon injury | Number of catheters/total PIVCs | 0/7,118 |
|  | Proportion, % (95% CI) | 0% |
|  | Time to removal*, median (IQR), hours | - |
|  | Incidence rate per 1,000 intravenous catheter days (95% CI) | 0 |
| Extreme compartment | Number of catheters/total PIVCs | 0/7,118 |
|  | Proportion, % (95% CI) | 0% |
|  | Time to removal*, median (IQR), hours | - |
|  | Incidence rate per 1,000 intravenous catheter days (95% CI) | 0 |
| Accidental removal | Number of catheters/total PIVCs | 61/7,118 |
|  | Proportion, % (95% CI) | 0.9 (0.7-1.1) |
|  | Time to removal*, median (IQR), hours | 25.0 (7.0-51.5) |
|  | Incidence rate per 1,000 intravenous catheter days (95% CI) | 3.7 (2.8-4.7) |
| Pain | Number of catheters/total PIVCs | 26/7,118 |
|  | Proportion, % (95% CI) | 0.4 (0.3-0.5) |
|  | Time to removal*, median (IQR), hours | 30.0 (17.6-92.5) |
|  | Incidence rate per 1,000 intravenous catheter days (95% CI) | 1.6 (1.0-2.2) |
| Redness | Number of catheters/total PIVCs | 9/7,118 |
|  | Proportion, % (95% CI) | 0.1 (0.07-0.2) |
|  | Time to removal*, median (IQR), hours | 74.1 (43.5-91.1) |
|  | Incidence rate per 1,000 intravenous catheter days (95% CI) | 0.6 (0.2-0.9) |
| Inappropriate insertion site | Number of catheters/total PIVCs | 20/7,118 |
|  | Proportion, % (95% CI) | 0.3 (0.2-0.4) |
|  | Time to removal*, median (IQR), hours | 49.7 (17.3-75.1) |
|  | Incidence rate per 1,000 intravenous catheter days (95% CI) | 1.2 (0.7-1.8) |
| Effusion around insertion site | Number of catheters/total PIVCs | 16/7,118 |
|  | Proportion, % (95% CI) | 0.2 (0.1-0.4) |
|  | Time to removal*, median (IQR), hours | 46.3 (32.2-87.2) |
|  | Incidence rate per 1,000 intravenous catheter days (95% CI) | 1.0 (0.5-1.5) |
| Others (skin trouble, line damage, anaphylaxis) | Number of catheters/total PIVCs | 2/7,118 |
|  | Proportion, % (95% CI) | 0.02 (0.008-0.1) |
|  | Time to removal*, median (IQR), hours | 110.6 (36.2-185.0) |
|  | Incidence rate per 1,000 intravenous catheter days (95% CI) | 0.12 (0-0.29) |

CI, confidence interval; CRBSI, catheter-related blood stream infection; IQR, interquartile range; PIVC, peripheral intravenous catheter

*time to removal means the duration between insertion and removal of the catheter.

**time to phlebitis means the duration between insertion of the catheter and detection of phlebitis.

**e-Table 7 Characteristics of PIVCs and outcomes according to insertion location**

|  | | Inserted in ICU | Inserted before ICU admission | |
| --- | --- | --- | --- | --- |
|  |  |  | ER | Others (OR, ward, outpatient) |
| Number of PIVC (n, %) | | 3,429 (48.2%) | 1,310 (18.4%) | 2,376 (33.4%) |
| Inserted Site (n,%) | |  |  |  |
| Upper arm | | 356/3,421 (10.4%) | 68/1,307 (5.2%) | 49/2,366 (2.1%) |
| Forearm | | 1,849/3,421 (54.1%) | 787/1,307 (60.2%) | 1,347/2,366 (56.9%) |
| Elbow | | 163/3,421 (4.8%) | 149/1,307 (11.4%) | 43/2,366 (1.8%) |
| Wrist | | 162/3,421 (4.7%) | 49/1,307 (3.8%) | 75/2,366 (3.2%) |
| Hand | | 507/3,421 (14.8%) | 207/1,307 (15.8%) | 800/2,366 (33.8%) |
| Lower leg | | 225/3,421 (6.6%) | 23/1,307 (1.8%) | 35/2,366 (1.5%) |
| Dorsal foot | | 137/3,421 (4.0%) | 17/1,307 (1.3%) | 15/2,366 (0.6%) |
| Others | | 22/3,421 (0.6%) | 7/1,307 (0.6%) | 2/2,366 (0.09%) |
| Catheter material | |  |  |  |
| PEU-Vialon* | | 1,087/3,429 (31.7%) | 279/1,310 (21.3%) | 753/2,376 (31.7%) |
| Polyurethane | | 978/3,429 (28.5%) | 3378/1,310 (28.9%) | 612/2,376 (25.8%) |
| Polyethylene | | 0/3,429 (0%) | 0/1,310 (0%) | 0/2,376 (0%) |
| Tetrafluoroethylene | | 1,292/3,429 (37.7%) | 568/1,310 (43.4%) | 835/2,376 (35.1%) |
| Others | | 72/3,429 (2.1%) | 85/1,310 (6.5%) | 176/2,376 (7.4%) |
| Catheter gauge (n,%) | |  |  |  |
| 14G | | 1/3,368 (0.03%) | 2/1,286 (0.2%) | 7/2,353 (0.3%) |
| 16G | | 74/3,368 (2.2%) | 22/1,286 (1.7%) | 290/2,353 (12.3%) |
| 18G | | 89/3,368 (2.6%) | 87/1,286 (6.8%) | 419/2,353 (17.8%) |
| 20G | | 888/3,368 (26.4%) | 728/1,286 (56.6%) | 848/2,353 (36.0%) |
| 22G | | 2,254/3,368 (66.9%) | 432/1,286 (33.6%) | 744/2,353 (31.6%) |
| 23G | | 0/3,368 (0%) | 0/1,286 (0%) | 1/2,353 (0.04%) |
| 24G | | 62/3,368 (1.8%) | 15/1,286 (1.2%) | 44/2,353 (1.9%) |
| Dressing (n,%) | |  |  |  |
| Chlorhexidine-impregnated dressing | | 0/3,396 (0%) | 0/1,298 (0%) | 1/2,347 (0.04%) |
| Sterile polyurethane dressing | | 3,327/3,396 (98.0%) | 1,277/1,298 (98.4%) | 2,238/2,347 (95.4%) |
| Non-sterile polyurethane dressing | | 60/3,396 (1.8%) | 9/1,298 (0.7%) | 16/2,347 (0.7%) |
| Gauze dressing | | 1/3,396 (0.03%) | 0/1,298 (0%) | 1/2,347 (0.04%) |
| Tape dressing | | 9/3,396 (0.2%) | 12/1,298 (0.9%) | 91/2,347 (3.9%) |
| Infection during catheterization (n,%) | | 803/3,429 (23.4%) | 271/1,310 (20.7%) | 193/2,376 (8.1%) |
| Duration of catheterization, median (IQR), hours | | 46.2 (21.3-82.9) | 41.7 (19.7-71.5) | 22.5 (17.8-47.3) |
| Removal in ICU (n, %) | | 2,102/3,421 (61.4%) | 780/1,306 (59.7%) | 822/2,374 (34.6%) |
| Catheter complications | |  |  |  |
| Phlebitis | Number of catheters/total PIVCs | 313/3,429 | 129/1,310 | 93/2,376 |
|  | Proportion (95% CI) | 9.1 (8.2-10.1) | 9.9 (8.4-11.6) | 3.9 (3.2-4.8) |
|  | Time to phlebitis**, median (IQR), hours | 37.0 (19.2-57.6) | 37.3 (16.9-59.9) | 28.2 (11.7-60.8) |
|  | Incidence rate per 100 intravenous catheter days (95% CI) | 7.8 (6.9-8.7) | 3.9 (3.2-4.6) | 1.1 (0.9-1.3) |
| CRBSI | Number of catheters/total PIVCs | 2/3,389 | 1/1,295 | 0/2,339 |
|  | Proportion (95% CI) | 0.06 (0.02-0.2) | 0.08 (0.01-0.4) | 0 (0-0) |
|  | Time to removal***, median (IQR), hours | 247.1 (12.75-481.5) | 558.3 (558.3-558.3) | - |
|  | Incidence rate per 1000 intravenous catheter days (95% CI) | 0.22 (0-0.52) | 0.33 (0-0.98) | 0 (0-0) |
| Catheter failure | Number of catheters/total PIVCs | 902/3,429 | 319/1,310 | 271/2,376 |
|  | Proportion (95% CI) | 26.3 (24.9-27.8) | 24.4 (22.1-26.8) | 11.4 (10.2-12.8) |
|  | Time to removal***, median (IQR), hours | 46.2 (21.3-82.9) | 41.7 (19.7-71.5) | 22.5 (17.8-47.3) |
|  | Incidence rate per 100 intravenous catheter days (95% CI) | 22.1 (20.7-23.6) | 9.3 (8.3-10.3) | 3.1 (2.7-3.4) |

CI, confidence interval; CRBSI, catheter-related blood stream infection; ER, emergency room; ICU, intensive care unit; IQR, interquartile range; OR, operation room; PIVC, peripheral intravenous catheter

*PEU-Vialon: polyetherurethane without leachable additives

**time to phlebitis means the duration between insertion of the catheter and detection of phlebitis.

***time to removal means the duration between insertion and removal of the catheter.

**e-Table 8 Characteristics of PIVCs and outcomes according to insertion location admission type**

|  | | Elective surgery | Emergency surgery | Medical emergency | |
| --- | --- | --- | --- | --- | --- |
| Number of PIVC (n, %) | | 2,504 (35.2%) | 1,280 (18.0%) | 3,334 (46.8%) |  |
| Location of insertion | |  |  |  |  |
| ICU | | 692/2,503 (27.7%) | 656/1,279 (51.3%) | 2,081/3,333 (62.4%) |  |
| ER | | 13/2,503 (0.5%) | 412/1,279 (32.2%) | 885/3,333 (26.6%) |  |
| Others (ward, OR, outpatient) | | 1,798/2,503 (71.8%) | 211/1,279 (16.5%) | 367/3,333 (11.0%) |  |
| Inserted Site (n,%) | |  |  |  |  |
| Forearm | | 1, 373/2,497 (55.0%) | 733/1,275 (57.5%) | 1,880/3,325 (56.5%) |  |
| Upper arm | | 59/2,497 (2.4%) | 85/1,275 (6.7%) | 329/3,325 (10.0%) |  |
| Elbow | | 26/2,497 (1.0%) | 94/1,275 (7.4%) | 235/3,325 (7.1%) |  |
| Wrist | | 83/2,497 (3.3%) | 53/1,275 (4.2%) | 150/3,325 (4.5%) |  |
| Hand | | 889/2,497 (35.6%) | 210/1,275 (16.5%) | 415/3,325 (12.5%) |  |
| Lower leg | | 33/2,497 (1.3%) | 54/1,275 (4.3%) | 196/3,325 (5.9%) |  |
| Dorsal foot | | 30/2,497 (1.2%) | 37/1,275 (2.9%) | 102/3,325 (3.1%) |  |
| Others | | 4/2,497 (0.2%) | 9/1,275 (0.7%) | 18/3,325 (0.5%) |  |
| Catheter material | |  |  |  |  |
| PEU-Vialon* | | 950/2,504 (37.9%) | 341/1,280 (26.6%) | 829/3,334 (24.9%) |  |
| Polyurethane | | 546/2,504 (22.5%) | 436/1,280 (34.1%) | 968/3,334 (29.0%) |  |
| Polyethylene | | 0/2,504 (0%) | 0/2,504 (0%) | 0/3,334 (0%) |  |
| Tetrafluoroethylene | | 855/2,504 (34.2%) | 430/1,280 (33.6%) | 1,410/3,334 (42.3%) |  |
| Others | | 135/2,504 (5.4%) | 73/1,280 (5.7%) | 127/3,334 (3.8%) |  |
| Catheter gauge (n,%) | |  |  |  |  |
| 14G | | 7/2,480 (0.3%) | 2/1,255 (0.2%) | 1/3,274 (0.03%) |  |
| 16G | | 315/2,480 (12.7%) | 58/1,255 (4.6%) | 13/3,274 (0.4%) |  |
| 18G | | 436/2,480 (17.6%) | 98/1,255 (7.8%) | 61/3,274 (1.9%) |  |
| 20G | | 833/2,480 (33.6%) | 456/1,255 (36.3%) | 1,175/3,274 (35.9%) |  |
| 22G | | 851/2,480 (34.3%) | 622/1,255 (49.6%) | 1,959/3,274 (59.8%) |  |
| 23G | | 0/2,480 (0%) | 0/1,255 (0%) | 1/3,274 (0.03%) |  |
| 24G | | 38/2,480 (1.5%) | 19/1,255 (1.5%) | 64/3,274 (2.0%) |  |
| Dressing (n,%) | |  |  |  |  |
| Chlorhexidine-impregnated dressing | | 0/2,477 (0%) | 0/1,267 (0%) | 1/3,299 (0.03%) |  |
| Sterile polyurethane dressing | | 2,358/2,477 (95.2%) | 1,236/1,267 (97.6%) | 3,250/3,299 (98.5%) |  |
| Non-sterile polyurethane dressing | | 29/2,477 (1.2%) | 10/1,267 (0.8%) | 46/3,299 (1.4%) |  |
| Gauze dressing | | 1/2,477 (0.04%) | 0/1,267 (0%) | 1/3,299 (0.03%) |  |
| Tape dressing | | 89/2,477 (3.6%) | 21/1,267 (1.7%) | 1/3,299 (0.03%) |  |
| Infection during catheterization (n,%) | | 76/2,504 (3.0%) | 258/1,280 (20.2%) | 933/3,334 (28.0%) |  |
| Duration of catheterization, median (IQR), hours | | 22.0 (17.8-42.0) | 46.8 (21.5-89.1) | 48.6 (21.9-79.5) |  |
| Removal in ICU (n, %) | | 748/2,500 (29.9%) | 758/1,278 (59.3%) | 2,199/3,325 (66.1%) |  |
| Catheter complications | |  |  |  |  |
| Phlebitis | Number of catheters/total PIVCs | 75/2,504 | 125/1,280 | 335/3,334 |  |
|  | Proportion (95% CI) | 3.0 (2.4-3.7) | 9.8 (8.3-11.5) | 10.1 (9.1-11.1) |  |
|  | Time to phlebitis**, median (IQR), hours | 21.0 (8.0-54.5) | 37.5 (18.7-64.7) | 37.3 (19.4-57.0) |  |
|  | Incidence rate per 100 intravenous catheter days (95% CI) | 0.84 (0.65-1.04) | 4.2 (3.5-5.0) | 8.2 (7.3-9.1) |  |
| CRBSI | Number of catheters/total PIVCs | 0/2,458 | 0/1,267 | 3/3,300 |  |
|  | Proportion (95% CI) | 0 (0-0) | 0 (0-0) | 0.09 (0.03-0.27) |  |
|  | Time to removal***, median (IQR), hours | - | - | 481.5 (12.8-558.3) |  |
|  | Incidence rate per 1000 intravenous catheter days (95% CI) | 0 (0-0) | 0 (0-0) | 0.34 (0-0.72) |  |
| Catheter failure | Number of catheters/total PIVCs | 233/2,504 | 318/1,280 | 941/3,334 |  |
|  | Proportion (95% CI) | 9.3 (8.2-10.5) | 24.8 (22.6-27.3) | 28.2 (26.7-29.8) |  |
|  | Time to removal***, median (IQR), hours | 22.0 (17.8-42.0) | 46.8 (21.5-89.1) | 48.6 (21.9-79.5) |  |
|  | Incidence rate per 100 intravenous catheter days (95% CI) | 2.6 (2.2-2.9) | 10.5 (9.4-11.7) | 22.5 (21.0-23.9) |  |

CI, confidence interval; CRBSI, catheter-related blood stream infection; ER, emergency room; ICU, intensive care unit; IQR, interquartile range; OR, operation room; PIVC, peripheral intravenous catheter

*PEU-Vialon: polyetherurethane without leachable additives

**time to phlebitis means the duration between insertion of the catheter and detection of phlebitis.

***time to removal means the duration between insertion and removal of the catheter.

References

1. Charlson ME, Pompei P, Ales KL, MacKenzie CR (1987) **A new method of classifying prognostic comorbidity in longitudinal studies: development and validation**.J Chronic Dis **40**: 373-383.

2. Seymour CW, Liu VX, Iwashyna TJ, Brunkhorst FM, Rea TD, Scherag A, Rubenfeld G, Kahn JM, Shankar-Hari M, Singer M, Deutschman CS, Escobar GJ, Angus DC (2016) **Assessment of Clinical Criteria for Sepsis: For the Third International Consensus Definitions for Sepsis and Septic Shock (Sepsis-3)**.JAMA **315**: 762-774.

3. Knaus WA, Draper EA, Wagner DP, Zimmerman JE (1985) **APACHE II: a severity of disease classification system**.Crit Care Med **13**: 818-829.

4. Le Gall JR, Lemeshow S, Saulnier F (1993) **A new Simplified Acute Physiology Score (SAPS II) based on a European/North American multicenter study**.JAMA **270**: 2957-2963.

5. Vincent JL, Moreno R, Takala J, Willatts S, De Mendonca A, Bruining H, Reinhart CK, Suter PM, Thijs LG (1996) **The SOFA (Sepsis-related Organ Failure Assessment) score to describe organ dysfunction/failure. On behalf of the Working Group on Sepsis-Related Problems of the European Society of Intensive Care Medicine**.Intensive Care Med **22**: 707-710.
